# Applications of next generation sequencing in the screening and diagnosis of thalassemia: A mini-review

**DOI:** 10.3389/fped.2022.1015769

**Published:** 2022-09-29

**Authors:** Syahirah Amnani Suhaimi, Ihsan Nazurah Zulkipli, Hazim Ghani, Mas Rina Wati Abdul-Hamid

**Affiliations:** PAPRSB Institute of Health Sciences, Universiti Brunei Darussalam, Gadong, Brunei

**Keywords:** NGS, targeted sequencing, WES, WGS, α-thalassemia, β-thalassemia, thalassemia programs

## Abstract

Thalassemias are a group of inherited blood disorders that affects 5–7% of the world population. Comprehensive screening strategies are essential for the management and prevention of this disorder. Today, many clinical and research laboratories have widely utilized next-generation sequencing (NGS) technologies to identify diseases, from germline and somatic disorders to infectious diseases. Yet, NGS application in thalassemia is limited and has just recently surfaced due to current demands in seeking alternative DNA screening tools that are more efficient, versatile, and cost-effective. This review aims to understand the several aspects of NGS technology, including its most current and expanding uses, advantages, and limitations, along with the issues and solutions related to its integration into routine screening and diagnosis of thalassemias. Hitherto, NGS has been a groundbreaking technology that offers tremendous improvements as a diagnostic tool for thalassemia in terms of its higher throughput, accuracy, and adaptability. The superiority of NGS in detecting rare variants, solving complex hematological problems, and providing non-invasive alternatives to neonatal diagnosis cannot be overlooked. However, several pitfalls still preclude its use as a stand-alone technique over conventional methods.

## Introduction

Thalassemia syndromes are a group of inherited blood disorders caused by mutations in the α- or β-globin genes (*HBA* or *HBB*), resulting in the formation of a reduced or abnormal globin peptide. The imbalanced globin synthesis leads to a decreased functional hemoglobin expression, which may result in variable clinical manifestations ranging from transfusion-dependent thalassemia major to mild forms of thalassemia intermedia (1). With a world estimation of 5–7% disease carriers and more than 2.4% annual birth rate, thalassemias are considered a vast socio and economic health burden, especially in highly prevalent regions (2, 3). Of the several types of thalassemias present, two of the most important forms are the α- and β-thalassemia; which have resulted mainly from deletions in the α-globin gene and point mutations, primarily small insertions/deletions (InDels) in the β-globin gene clusters respectively (4). Although the prognosis for thalassemias has improved substantially in the last few decades with proper adjustments in treatment and management protocols, patients with severe forms still require lifelong care, which is cumbersome, costly, and often results in detrimental secondary comorbidities (5, 6). For this reason, researchers have encouraged the prevention of new cases of thalassemias through screening programs. This is crucial in maintaining an ideal annual birth rate of severe thalassemia and reducing the cost of lifelong support for these patients (7, 8).

**FIGURE 1:**
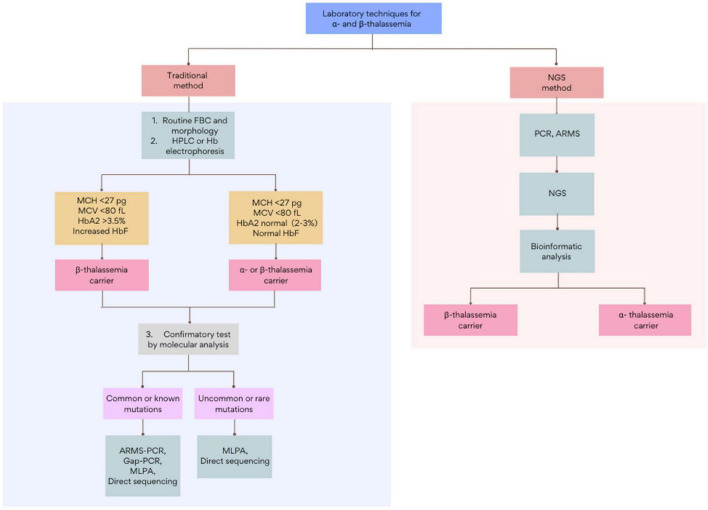
The general workflow for the screening and diagnosis of α- and β-thalassemia using traditional and NGS-based methods. Figure adapted from Chen et al. (28) and Nigam et al. (55). FBC, full blood count; HPLC, high-performance liquid chromatography; ARMS, amplification refractory multiple sequencing; PCR, polymerase chain reaction; MLPA, multiplex ligation-dependent probe amplification.

Current thalassemia assessments follow a three-step workflow that begins with a full blood count (FBC) and red cell morphology. The initial detection of thalassemia is based on low hemoglobin levels and abnormal red cell indices: mean corpuscular volume (MCV) and mean corpuscular hemoglobin (MCH). This is followed by biochemical analysis using Hb electrophoresis, high-performance liquid chromatography (HPLC), or capillary electrophoresis (CE) (9). Subsequently, based on the initial results, routine genetic testing such as Gap-PCR, Reverse dot blot (RDB), multiplex ligation-dependent probe amplification (MLPA), or direct Sanger sequencing, among others, will be adopted as a confirmatory test (Figure 1). Though these methods are the gold standards in thalassemia investigation, each test is highly labor-intensive. Furthermore, with over 1,530 genomic mutations identified to date, the interaction of disease variants with other abnormal hemoglobin genotypes or modifiers genes further complicates the interpretation of thalassemia (10–12). Among other limitations, the inadequacy of traditional methods to accurately diagnose uncommon mutations necessitates an alternative DNA screening tool (13, 14). Over recent years, tremendous improvements in sequencing technology and the bioinformatic understanding of genomic data have led to the increased clinical applications of next-generation sequencing (NGS). This breakthrough technology has provided an enhanced diagnostic yield and showed a drastic reduction in the cost and turnaround times for genome analysis, making it a suitable and reliable alternative technique for thalassemia detection. With genetic information becoming more precise, clinicians can give thorough explanations to help clarify complex clinical pictures. This review will report and discuss the applications and recent advances of NGS in the screening and diagnosis of thalassemia. This paper will also provide a compendium on the current challenges in the implementation of NGS in thalassemia and discuss the future directions in applying NGS in clinical settings.

### Overview of next-generation sequencing technology

After decades of “omics” expansion, NGS has become a better alternative to the first-generation Sanger sequencing in terms of accuracy, robustness, and handling (15). NGS has allowed relatively easier analyses of millions of short sequence reads which covers a broader spectrum of mutations, including InDels, single-nucleotide polymorphism (SNP), and large copy-number variations (CNV) in a single tube reaction (16). Compared to the previous technology, NGS is more feasible and efficient as a first-tier diagnostic tool as it reduces the need for multiple primary methods that raise the risk of unwanted sample mix-ups and contaminations. With this advancement, clinicians can now make a timely and comprehensive diagnosis which aids in informed genetic counseling and personalized medicine (17). Furthermore, with continuing refinements of sequencing platforms, the price of NGS is becoming more affordable over time, from USD5,292.39/Mb in 2001 to less than USD0.01/Mb in 2021 (17, 18).

Next-generation sequencing technologies have been used in various implementations, but the most common utilizations that will be reviewed here include whole-exome sequencing (WES), whole-genome sequencing (WGS), and targeted capture sequencing (TCS). The sequencing protocols of NGS are similar for all applications, irrespective of the sequencing platforms. The initial step includes fragmentation of DNA into random lengths followed by amplification using polymerase chain reaction (PCR) or hybridization approaches. WGS amplifies the entire gene in the genome while WES and TCS amplify only the protein-coding regions (exons) or a group of selected genes, respectively.

Amplified products are then loaded into a chosen sequencing platform to generate millions of short-read sequences, and the sequencing data are processed and analyzed in various bioinformatic pipelines (9, 19, 20). In short, the downstream analysis involves the reads mapped to a known reference genome followed by the detection and classification of variants in compressed data outputs called variant calling format (VCF) files (21). Selecting the appropriate NGS method and the bioinformatic tool is crucial in enhancing read depth (coverage), specificity, and sensitivity (22). Most WGS experiments would display a 30× coverage which is usually adequate to detect most known variants but inadequate to identify rare mutations. Conversely, since WES targets only 2% of a genome, a single region can be annotated more, increasing the coverage to 100×. When TCS is utilized, more focused time is given to each interrogated gene; hence coverage could spike to 500–1000× to produce a more high-quality variant calling (19, 23).

### Applications of next-generation sequencing in thalassemia

#### Next-generation sequencing in the molecular screening of thalassemia carriers

Mass screening strategies and appropriate genetic counseling are mandated for people at different life stages in high-risk populations to reduce the births of thalassemia major babies (24). In highly prevalent regions, carrier screening is mainly aimed toward the general population at random. When an individual is suspected with thalassemia, they are subsequently referred for further hematological and biochemical testing. This practice helps for early determination of carriers and generation of a population prevalence density. A number of studies have been carried out to validate the NGS-based approaches in population screening, and a majority of them reported improved detection rates compared to standard methods (25, 26). The use of NGS was shown to not only help detect missed thalassemia carriers but also identify unknown mutations that are typically undetected by routine analysis. Moreover, combining NGS with conventional molecular tools such as Gap-PCR has also been demonstrated to optimize screening and improve diagnostic yield (26, 27). Newer platforms such as Ion Torrent have also been proven to have 100% consistency to routine methods, but with more flexibility than other NGS protocols for medium-sized laboratories, which is appealing for thalassemia screening in endemic countries (28).

An equally important screening strategy is directed toward prospective parents. For known carriers, the screening of their partner can aid in risk assessment through genetic counseling. Throughout the decades countries like Sardinia, Cyprus, and Israel have successfully reduced thalassemia burden through these mandatory premarital screenings (8). Notably, the use of NGS has greatly enhanced the effectiveness of current thalassemia prevention compared to routine methods. He et al. (13), for instance, reported higher detection of thalassemia carriers using TCS (49.5%) than conventional approaches (22%). Interestingly, most of the missed individuals were silent α-thalassemia carriers, where more than 90% had the α^3.7^/αα genotype. These variants were missed during the first-tier assessments, which terminates them from downstream analysis.

Furthermore, 47 carriers of the large –*^SEA^* deletion, the second most abundant α-thalassemia mutation in China, were also missed by routine Hb electrophoresis. This increases the risk of HbH formation or even Hydrops fetalis if the other partner is also a carrier of the α^+^ or α^0^ determinants, respectively (29, 30). Another group that designed the TCS gene panel to cover all the eight globin genes and four gene modifiers (KFL1, BCL11A, HBS1L, and MYB) to screen newlyweds also demonstrated similar enhanced detection rates by detecting 35 at-risk couples initially missed by routine analysis (25). Similarly, the combined NGS and Gap-PCR method are also reported to significantly reduce false-negative results and misdiagnosis in couple carriers (27).

Recently, NGS has also been applied in newborn screening (NBS) programs in regions of high prevalence (31). Due to the rapid nature of disease progression in neonates, diagnostic yield and speed are fundamental aspects of obtaining a high clinical impact (32). NGS methods allow the early recognition of affected infants as early as their first 24–72 h of life (33). Identified at-risk newborns can then be monitored closely for the development of anemia and start transfusion at the appropriate time, minimizing the risk of complications (12). Using the combined NGS method, Tan et al. (34) reported a higher detection rate compared to the traditional NBS method which had 65 missed thalassemia carriers. More importantly, 3 out of 10 β-thalassemia major babies identified with NGS presented with clinical symptoms during the follow-up stage and were given timely interventions. This minimized the risk of patient health deterioration and improved their overall quality of life (34). The integration of high-throughput sequencing with conventional dried-blood spot (DBS) not only enables a faster turnaround time, but also gives a more reliable result for the parents, enabling them to be emotionally prepared for their sick child (35, 36).

#### Next-generation sequencing in the molecular diagnosis of thalassemia

Accurate identification of disease severity is a prerequisite for a proper treatment selection. This is especially important for patients with atypical clinical presentations or when standard genotyping methods produce inconclusive diagnoses. One study (37) examined a rare case of severe anemia with accompanying splenomegaly in an IVS-I-110 (G > A) thalassemia carrier (β^+^/β*^wt^*). After deep sequencing with WES, the analysis revealed an α-globin triplication αα/ααα*^anti3.7^* variant found coinherited with the heterozygous β-globin mutation rendering it to aggravate the trait phenotype into thalassemia intermedia (TI) (37). With this redefined diagnosis, clinicians can make an informed and timely decision for treatment selections, including subsequent blood transfusion and iron overload assessments.

In other cases, NGS also enables the identification of at-risk family members, which facilitates the institution of counseling and treatments early in the presymptomatic phase without the need for troublesome linkage-based methods (38). This is demonstrated by Sabiha et al. (39), who, while validating the diagnosis of a rare homozygous A > AC/AC insertion mutation from a transfusion-dependent β-thalassemia affected child with NGS, also identified all the healthy family members as β-thalassemia carriers of the same synonymous mutation. With a single test, not only does this safeguard from passing the deleterious mutation, but it also helps national thalassemia programs to confirm the competency of the rare frameshift variant to cause β-thalassemia major. Moreover, in cases of compound polymorphism such as HbS/β-thalassemia, diagnosis can be notoriously challenging because of the overlapping phenotypic characteristics. Adekile and group (40) reported a higher diagnostic rate of HbS/β-thalassemia using the NGS-based approach (29.4%) than routine HPLC (27.7%). Although the difference was minor, most of the misdiagnosed individuals carried IVS-I-5 (G > C) and IVS-I-6 (C > T), which are the major β^+^ determinants for βTI (41).

A more exciting milestone of NGS is the introduction of non-invasive prenatal diagnosis (NIPD). This method facilitates the prevention of thalassemias through termination of affected pregnancies using low abundance cell-free fetal DNA (cffDNA) present in maternal circulation (42). This fetal DNA source can be obtained through venipuncture, which is safer than previously described amniocentesis and chorionic villi sampling. Moreover, NGS-based approaches combined with conventional relative haplotype dosage (RHDO) analysis is a technique which is increasingly adapted into the NIPD protocol due to its feasibility and low-cost (43). NGS provides efficient means of constructing parental haplotypes to assess fetal genotypes, i.e., by removing the needs of complicated haplotypic block/proband analysis (44). Although errors from maternal background contaminations still remains an issue, the combined NGS-RHDO method has been demonstrated to provide accurate fetal risk assessments concurrent with the results obtained through invasive prenatal diagnosis procedures (45–47). Further, a recently developed NGS-based NIPD by Yang et al. called the cffDNA barcode enabled single molecule test (cfBEST) is a counting system to quantitatively deduce maternal and fetal genotypes (48). In their blind validation study, cfBEST achieved over 99% in both sensitivity and specificity, making it a promising system for large-scale NIPD in the future.

Alternatively, several researchers have also developed NGS-based preimplantation genetic diagnosis (PGD), which gives at-risk couples with severe thalassemia babies opportunities to get pregnant with a healthy child without the need for termination. This is a preferred method for individuals with medical contraindications to abortions or those who are generally against the idea of termination due to ethical or religious beliefs. It also saves the mother from physiological stress post-surgery and eliminates maternal death risk due to procedural complications (49, 50). Although several existing techniques are being utilized for thalassemia PGD, there are still high error rates owing to the interruptions by allele drop-outs (ADOs) and DNA contaminations (51). Thus, to optimize the sensitivity and efficiency of PGD, several studies have remodeled the procedure with NGS-based techniques. Chen et al. (52) combined WGS with linkage analysis to screen blastocysts from an HbH at-risk couple and compared it to Gap-PCR. Their results show the superiority of NGS for PGD in detecting the -α^3.7^ and –*^SEA^* genotypes without the occurrence of ADOs, which is remarkable compared to the 80% ADOs present when using Gap-PCR. Similarly, a large PGD study that synchronously screened both α/β-thalassemia with other aneuploidies in 112 blastocysts showed an ideal diagnostic rate which resulted in the birth of 11 healthy babies from 12 at-risk couples who, in the majority, have a history of recurrent spontaneous miscarriages (53).

## Discussion

Although medical advances have transformed the outlook of severe thalassemia patients, their quality of life is still restricted by physiological and social constraints. Their reliance on regular blood transfusions and iron chelation therapies to prolong survival hampers daily activities and risks the development of life-threatening complications (54). Moreover, these treatments can also bring a substantial economic burden to the patients and the health sector, demanding immediate prevention strategies (55). Unlike other monogenic diseases, thalassemia detection depends greatly on routine hematological techniques. Unfortunately, besides being laborious, these methodologies are rife with ambiguities, particularly in determining carrier states or solving complex compound heterozygotes cases. As carriers are predominantly asymptomatic, their atypical hematological findings would cause them to be undetected during screening (14). Consequently, if their partner is also a disease carrier, these missed individuals would be at risk of transmitting the pathogenic gene to their offspring, causing severe thalassemia.

The limitations of conventional red cell indices and HPLC as predictors of carrier status have been shown in several publications. Two reports have estimated that around 20–30% of individuals with normal or borderline HbA_2_ were actually positive for β-thalassemia trait (βTT), and up to 37% of βTT individuals can be missed in screening (56, 57). This missed diagnosis is primarily the result of compound inheritance with iron-deficiency anemia (IDA) and coinheritance with α-thalassemia (13, 58). Moreover, thalassemia carriers are also easily misdiagnosed as IDA due to similarities in phenotypic characteristics, leading to unnecessary iron supplementation. This can cause overload later in life, leading to complications such as pulmonary hypertension and thrombosis (59). Another major setback of conventional methods is their inability to detect rare or novel variants. One study reported that up to 10% of patients with uncommon mutations could be missed by routine RDB and MLPA (60). These problems, therefore, raise demands for alternative first-tier molecular tools for thalassemia programs.

The implementation of NGS in thalassemia has been widely accepted as it offers a more accurate and simplified diagnostic workflow (Figure 1 and Table 1) (61). This, in turn, gives a more comprehensive and timely diagnosis than conventional methods. Moreover, its feasibility in detecting unknown genetic sequences and rare population-specific variants is an added feature that dominates it over other molecular methods for thalassemia programs. Furthermore, given that most of the current NGS cost per run in a 96-well plate is less than USD$1500, it is possible to simultaneously sequence multiple samples at the cost of approximately USD$15–25, making them as affordable as regular thalassemia analysis (27, 62).

**TABLE 1:** List of studies using NGS in thalassemia screening and diagnosis from 2017 to 2022.

Reference	Method	Study design	Country	Year	Key outcomes
Shang et al. (25)	NGS (TCS) vs. TM	Population and Premarital screening	China	2017	12.1% variants were missed by TM, with additional 35 at-risk couples being identified by NGS
Zhang et al. (26)	NGS (TCS) + Gap-PCR vs. TM	Population screening	China	2019	2.88% of carriers were missed by TM, and an additional five novel mutations were identified by the combined NGS method
Chen et al. (28)	NGS (TCS) vs. TM	Population screening	China	2020	NGS method was reported to have 100% consistencies to TM, and this protocol is reportedly best suited for medium-sized laboratories
He et al. (13)	NGS (TCS) vs. TM	Premarital screening	China	2017	NGS method detected a higher carrier rate (49.5%) compared to TM (22%), and almost 90% of missed carriers had α^37^/αα genotype
Zhao et al. (27)	NGS (TCS) + Gap-PCR vs. TM	Premarital screening	China	2020	The combined NGS method detected seven additional rare mutations which were not detected by TM
Tan et al. (34)	NGS (TCS)	Newborn screening	China	2021	NGS method detected 65 carriers that were missed by TM. 3/10 of β -thalassemia major babies identified by NGS showed clinical symptoms during follow up stage and were given early interventions.
Sabiha et al. (39)	NGS (TCS)	Diagnostic test	Pakistan	2020	NGS method confirmed rare P-thalassemia diagnosis in an affected child and simultaneously diagnosed all healthy members as carriers
Sterinberg-Shemer et al. (37)	NGS (WES)	Diagnostic test	Israel	2017	NGS method corrected and re-diagnosed a thalassemia carrier as thalassemia intermedia
Adekile el al. (40)	NGS (TCS)	Diagnostic test	Kuwait	2021	NGS method gave a higher diagnostic rate (29.4%) compared to TM (27.7%)
Jiang et al. (43)	NGS + TM	Non-invasive prenatal diagnosis	China	2021	The combined NGS method correctly classified fetal status in 12/13 families
Erlich et al. (45)	NGS (TCS)	Non-invasive prenatal diagnosis	USA	2022	NGS method showed correct fetal diagnosis in 9/10 cases with one inconclusive result resulting from direct maternal contaminations
Chen et al. (52)	NGS (WGS) + linkage analysis	Preimplantation Genetic Diagnosis	China	2017	The combined NGS method reported lower ADOs (0%) compared to TM (80%), resulting in the birth of a healthy infant
Chen et al. (53)	NGS (WGS)	Preimplantation Genetic Diagnosis	China	2020	NGS method showed an ideal diagnostic rate and the birth of 11/12 healthy babies with no reports of miscarriages

NGS, next-generation sequencing; TCS, target capture sequencing; WES, whole-exome sequencing; WGS, whole-genome sequencing; TM, traditional methods; NBS, newborn screening; ADOs, allele drop-outs.

Despite its immense qualities and potential, several handicaps have delayed its use as a stand-alone technique in routine clinical settings. Among the significant pitfalls of NGS is the generation of short-read sequences (63). This problem causes occasional misinterpretations, especially with large deletions in the *HBA* gene cluster due to the highly homologous *HBA1* and *HBA2* subunits. Another limitation of NGS is its nature to erroneously map sequences in guanine-cytosine (GC)-rich regions. Thus, mutations in these GC regions will be missed, resulting in false-negative results. Compared to its counterparts, such as MLPA and Comparative Genomic Hybridization (CGH) analysis, NGS has been reported to be less accurate in these cases. Therefore, best practice recommends that NGS to always be paired with Gap-PCR to compensate for this shortcoming, and Sanger sequencing to remain a mandatory confirmatory test (64). Further, the advent of long molecule third-generation sequencing technology may also offer a similar solution by directly reading the entire length of the gene sequence with no apparent GC preference (65, 66). Additionally, since NGS is moving rapidly into diagnostic laboratories, and novel variants are progressively added into the HbVar database, new variants of uncertain significance (VUS) will definitely be encountered (31). Therefore, the next challenge that lies is in accurately translating these data to ensure meaningful clinical interpretation and counseling until more information becomes available (67, 68).

However, Regardless of the abovementioned limitations, NGS is still a valuable addition that fills the gap of conventional methods in identifying the mutational landscape of thalassemias. Its revolutionary applications in certain circumstances, such as large-scale screening, detecting unknown and rare variants, solving complex hematological cases, and being an alternative to invasive prenatal diagnosis, cannot be overlooked. However, until improvements are made, low-cost routine techniques will remain the gold standard in thalassemia detections, especially in endemic regions. The advent of NGS in clinical settings navigates toward prompt implementation of a standardized protocol in both sequencing and bioinformatic pipelines to ensure consistency between laboratories (69). Moreover, as NGS platforms continue to evolve, further research should validate and review the clinical guidelines in the variable NGS applications in thalassemia to enhance its efficiency and provide harmonization in global thalassemia programs.

## Conclusion

Next-generation sequencing applications in thalassemia have remained vastly understudied compared to other genetic disorders. However, this technology has, without doubt, marked a turning point in the diagnosis of thalassemia syndromes, and the findings thus far have provided a glimpse into the era of genomic medicine and the infinite possibilities offered by NGS. Although there are still several limitations to NGS methods, it remains an impressive tool that can be anticipated to be incorporated into routine clinical practice for thalassemia. With the cost of NGS being increasingly affordable, this technology is likely to be scalable into thalassemia programs to enable better diagnosis and offer personalized treatments in the near future. However, at present, it cannot be denied that similar outcomes can also be obtained using standard techniques.

## Author contributions

MA-H conceptualized the original title. SS completed the initial literature review and initial manuscript draft. IZ and HG provided the critical review. All authors contributed to the article and approved the submitted version.
